# Policy networks and competing interests in the development of the Mexican sugar-sweetened beverages tax

**DOI:** 10.1136/bmjgh-2023-012125

**Published:** 2023-10-09

**Authors:** Angela Carriedo, Paul Cairney, Simón Barquera, Benjamin Hawkins

**Affiliations:** 1 World Public Health Nutrition Association, London, UK; 2 Division of History, Heritage, and Politics, University of Stirling, Stirling, UK; 3 Centre of Research in Nutrition and Health, National Institute of Public Health, Cuernavaca, Mexico; 4 MRC Epidemiology Unit, University of Cambridge School of Clinical Medicine, Cambridge, UK

**Keywords:** Health policy, Public Health, Nutrition

## Abstract

**Introduction:**

Sugar taxes threaten the business models and profits of the food and beverage industry (F&BI), which has sought to avert, delay or influence the content of health taxes globally. Mexico introduced a sugar-sweetened beverage (SSB) tax in 2014 and other regulatory measures to improve population diets. This paper examines how policy networks emerged within and affected the development and implementation of the Mexican SSB tax.

**Methods:**

This qualitative study analyses 31 interviews conducted with key stakeholders involved in the soda tax policy process and 145 documents, including grey literature and peer-reviewed literature. The policy network approach was used to map contacts, interconnections, relationships and links between the state, civil society and commercial actors involved in the SSB tax. These findings were used to examine the responsiveness, participation and accountability of the soda tax policy formulation.

**Results:**

Complex interconnections were identified between state and non-state actors. These included advisory relationships, financial collaborations and personal connections between those in high-level positions. Relationships between the government and the F&BI were not always disclosed. International organisations and academics were identified as key financial or technical supporters of the tax. Key governance principles of participation, responsiveness and accountability were undermined by some of these relationships, including the participation of non-state actors in policy development and the powerful role of the F&BI in evaluation and monitoring.

**Conclusion:**

This case study exemplifies the importance of links and networks between actors in health policymaking. The F&BI influence endangers the primary aim of the SSB tax to protect health. The identified links highlight the normalisation of connections among actors with competing aims and interests toward health, thereby jeopardising attempts to tackle obesity rates.

**WHAT IS ALREADY KNOWN ON THIS TOPIC**

- Policy network analysis examines the links and relationships between actors to explain social actions and policy changes and to understand their implications.
- Such relationships can shape the formulation of policies such as health taxes and affect the policy process’s responsiveness, openness and accountability.

**WHAT THIS STUDY ADDS**

- Using the principles of policy network analysis, we identified how policy networks influenced the agenda setting and policy formulation of the Mexican SSB tax.
- This study sheds light on the politics of implementing health taxes, identifying networks of power and influence that shaped the resulting taxation policy.

**HOW MIGHT THIS STUDY AFFECT RESEARCH, PRACTICE OR POLICY**

- Identifying these relationships will enable researchers and advocates to seek to improve norms and practices within the policymaking process, for example, the introductions of proper declarations of interest and ruling out certain practices.
- The actions of issue networks and interest groups involved in health taxation policies need to be carefully considered as they may be relevant to, or inform, debates on other policy areas and health issues.

## Introduction

Non-communicable diseases (NCDs) are the leading cause of death and disability worldwide, killing approximately 41 million people annually.^1^ National authorities globally have implemented various countermeasures, including the introduction of taxes on sugar-sweetened beverages (SSBs).^2^ These reduce consumption of unhealthy products, promote healthier alternatives (eg, water) and provide funding for health programmes.^3^ SSB taxes have been implemented in more than 50 countries in different forms^4^ but have been constantly challenged by the food and beverage industry (F&BI) and its allies.^5–7^ Sugar taxes threaten the business models, sales and profits of sugar producers, and the F&BI has acted strategically to avert, delay or influence the content of fiscal measures through similar strategies to those employed by the tobacco and alcohol industries.^8 9^,^10–13^ The F&BI political activities in low and middle-income countries have been much less researched than tobacco’s political activities in the same context.^14–16^ Analysis of how commercial interests interfere with the implementation of SSB taxes, thus addresses a broader gap in the global health policy literature.^17^

Mexico is experiencing an obesity epidemic and high rates of diabetes^18^ and has sought to address this through a range of policy instruments, including SSB taxes. In 2013, the Mexican government implemented the ‘*National Strategy to Prevent and Control Obesity and Diabetes*’ (*Estrategia Nacional para la Prevención y el Control del Sobrepeso, la Obesidad y la Diabetes*; ENPCSOD).^19^ It set out three strategic pathways for regulation: (1) restricting food marketing exposure to children, (2) implementing a front-of-package food labelling and (3) having an SSB tax and a snack tax. In January 2014, the excise tax (Impuesto Especial Sobre Producción y Servicios; IEPS) of 1 Mexican Peso per litre of SSBs (including carbonated beverages, flavoured drinks containing sugar and powders and concentrates to prepare sugary drinks) came in effect. In 2015, the Ministry of Health (MoH) launched the Mexican Observatory of Non-Communicable Diseases (‘Observatorio Mexicano de Enfermedades No-Transmisibles’; OMENT): a multistakeholder and multisectoral platform, which aimed to set indicators for measuring the impact of the strategy, and its monitoring and evaluation processes, which included food industry actors and civil society organisations.^20^

Recent decades have seen an increasing shift towards coregulatory and partnership-based health policy responses involving civil society actors and the private sectors.^21–23^ As Kenis and Schneider comment, there has been a ‘blurring of boundaries’ between the public and private sectors.^24^ These approaches have been promoted by international organisations to address NCDs and to deliver and progress on the Sustainable Development Goals^25^ but have been heavily criticised by others as ineffective and counter-productive due to the conflicts of interest involved with industry engagement.^25 26^ The ‘decentring’ of the state in contemporary forms of governance has also given rise to a range of concepts and analytical frameworks within the fields of political science and policy studies, which seek to understand and explain the influence of ‘policy communities’,^27^ ‘iron triangles’,^27 28^ ‘issue networks’^29^ and ‘advocacy coalitions’^30^—which can be captured under the broader rubric of ‘policy networks’—over the definition of policy problems and the development of interventions to address them. These approaches differ in how they conceptualise policy networks, which can be more open and fluid or more closed and stable in terms of their membership and structure. The former is associated with greater internal contestation given the larger number of actors involved, while the latter is characterised by a greater homogeneity of interests among a smaller set of actors.^31^

In this context, it is essential to study the role of non-state actors—and the emergence of policy networks between state, civil society and commercial actors—in the development of novel policies such as SSB and other health taxes.^32–34^ Here, we define policy networks as a set of formal and informal linkages between governmental (state) and other (non-state) actors. Some scholars identify coalitions based on shared beliefs, often with one coalition able to translate its beliefs into policy. Others identify close relationships based on resource sharing (with group resources ranging from representativeness, to being essential to policy delivery, to providing material resources to policymakers) and a shared definition of the problem (some policy communities). And some focus on networks related to strategy at the ‘centre’ of government, more local policy delivery, or both.^31^ Policy network analysis (PNA) examines the contacts, relationships, links, interdependence and dynamics as part of structural factors^35–37^ or of agency that each of the actors and the relationships involved in policy formulation and decision-making have.^35^ These have implications for the principles of good governance—accountability, transparency and responsiveness^38^—which have helped to guide and analyse health policies and initiatives globally.

This article examines how policy networks emerged within and affected the agenda-setting and policy formulation of the Mexican SSB tax. We analyse the interconnections and links (networks) between actors involved in Mexico’s 2014 SSB to illustrate how they played for or against the policy change: and to have insights into the governance principles of responsiveness, participation and accountability while the policy was discussed and passed. In doing so, this paper seeks to address wider gaps in the literature about how networks can affect agenda setting and policy formulation in LMICs and to build on the recent call by public health researchers to understand better corporate power and the factors facilitating or undermining this power.^39^

## Methods

**Figure 1:**
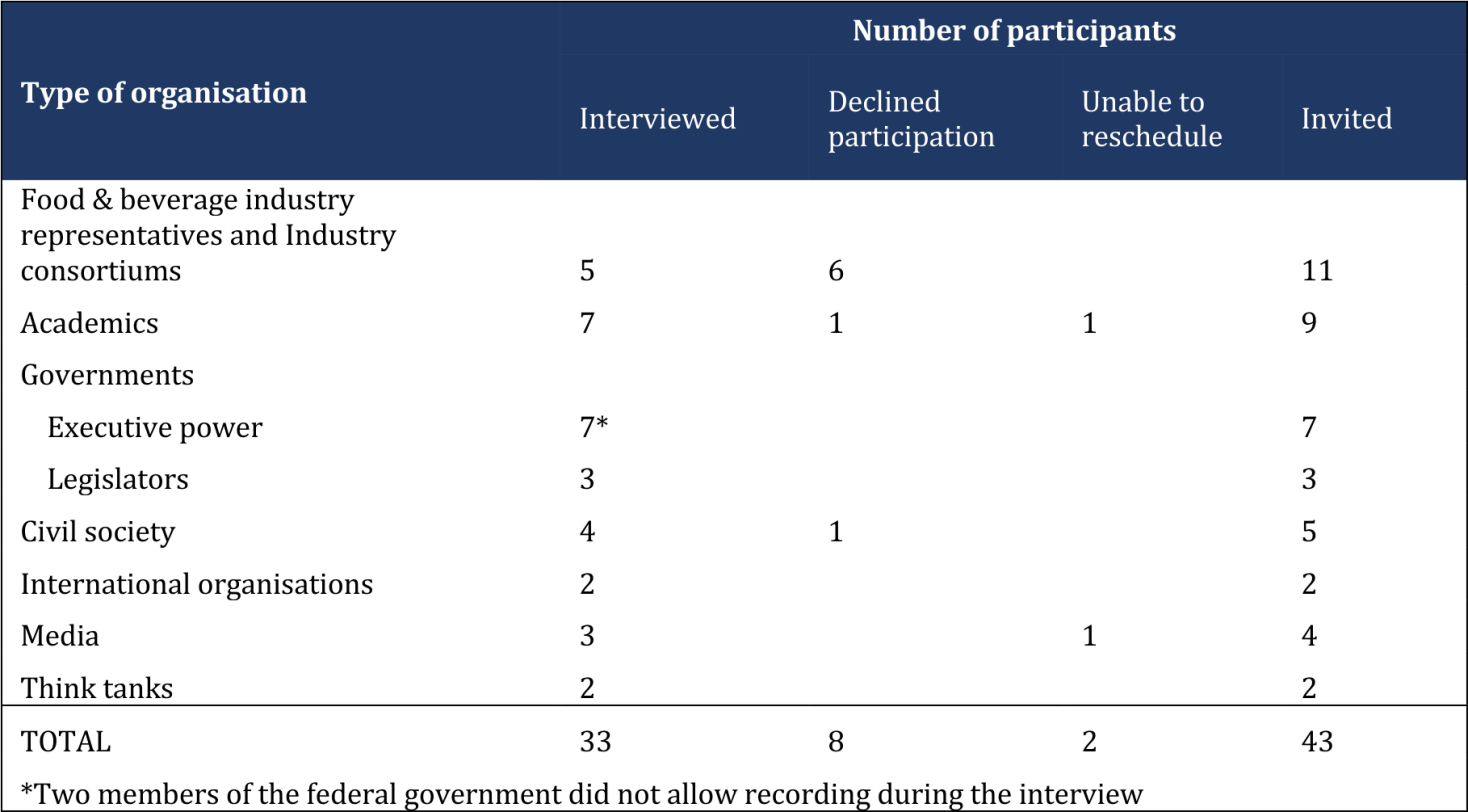
Actors interviewed.

This article emerges from a qualitative PNA of the Mexican SSB tax debates. The first author conducted 31 interviews with key stakeholders involved in the policy process and included government, civil society, the F&BI, marketing and media experts and academics (from October 2014 to December 2014) identified via a stakeholder analysis^40^ and presented in figure 1. These interviews were triangulated with a documentary analysis of 145 documents, including government, industry and civil society reports, peer-reviewed journals, and media outputs (from November 2015 to March 2016).

### Participant selection

**Figure 2:**
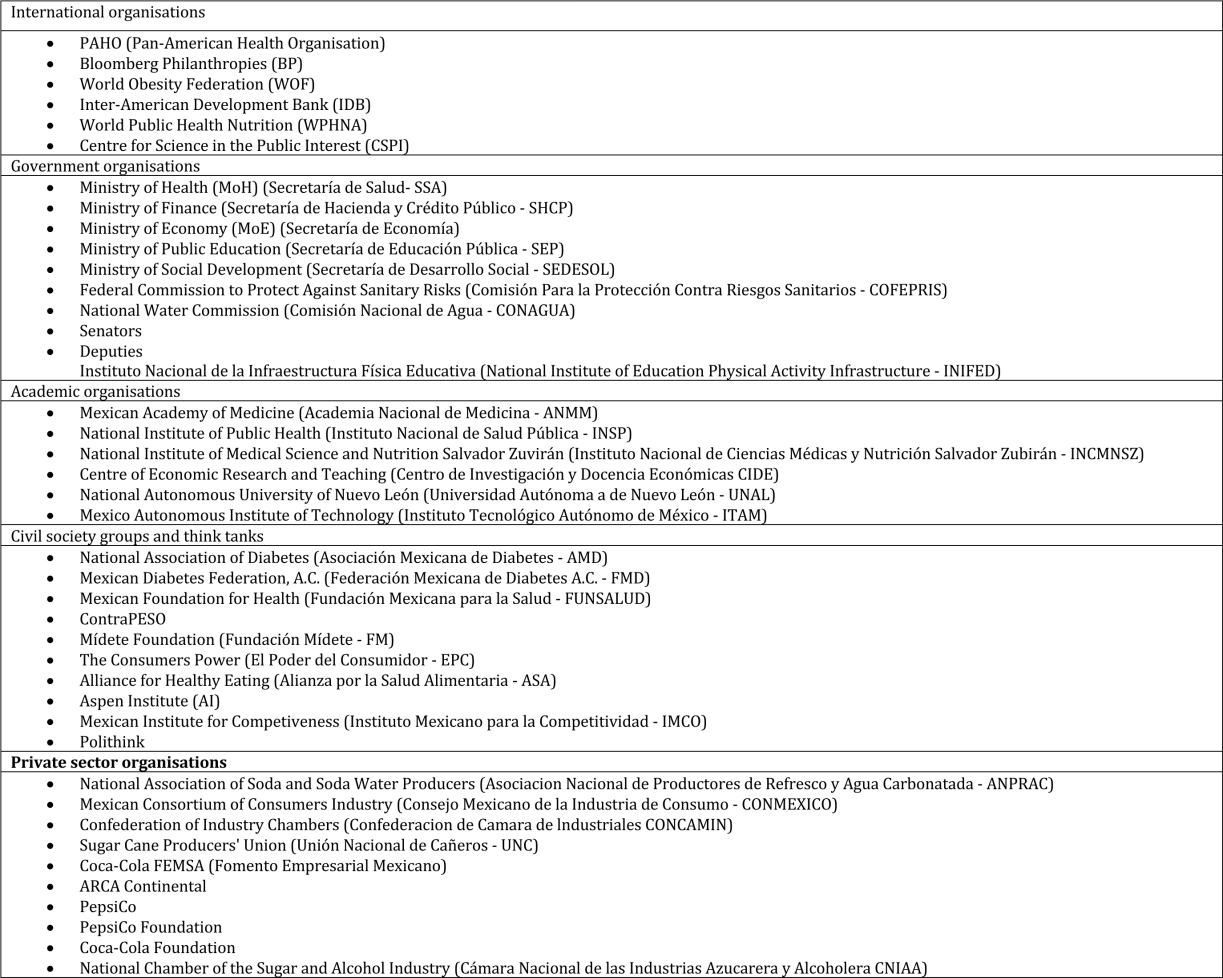
Organisations identified as being involved in the soda tax policy.

The interview sample frame was determined purposively after conducting a literature review and stakeholder analysis to include respondents from all disciplines and sectors involved in the SSB tax policy. After conducting the first set of interviews, a snowballing technique was applied to include more actors.^41^ Figure 2 shows the list of actors identified to be involved in the SSB tax. The stakeholders were mapped^42^ to identify all actors according to their power-interest at the time (2013). In this case, power measures their influence over the policy and the degree of their ability to block or change the policy. Interest is the degree to which they are likely to be affected by the policy change.^42^ This allowed the sample to be as inclusive as possible and reduce bias in the researcher’s judgement.^43^ Figure 2 shows the list of actors identified as involved in the SSB tax.

### Participants and public involvement

Participants were not involved in developing the research questions or data analysis. The findings of this research emerge from a reflexive process of analysing data provided by participants via interviews. The results of this research will be made available to them.

### Document selection

Documents were searched in the websites of organisations identified in the stakeholder mapping, followed by a search of documents using a combination of keywords in Spanish and in English like ‘impuesto’, ‘gravamen’, ‘IEPS’, ‘reforma fiscal’, ‘levy’, ‘tax’ and ‘bebidas azucaradas’, ‘refrescos’ ‘sodas’, ‘sugary drinks’, ‘sugar sweetened beverages’ in Google Scholar, Web of Science and the websites of organisations involved in the policy. The time frame was defined a priori as 5 years, from January 2011 to December 2015. This period includes the first attempts by legislators to include an obesity policy on the government’s agenda and the fiscal reform in 2013, when the soda tax became effective. The 2 years following the fiscal reform are also included in the analysis to document the main reactions and positions following the policy change. Documents were then screened according to the relevance of our objectives.

### Analysis

The interviews were transcribed *verbatim* in Spanish, uploaded to NVivo software V.11 and coded. An iterative analysis was applied to define the codes and themes. Some codes were first defined deductively,^44 45^ and other themes emerged from the data. The coding frame (online supplemental material table 1) was discussed among coinvestigators (BH, ACL) to reach consensus, and 20% of the interview transcripts in Spanish were read and reviewed by one coinvestigator (BH). Triangulation between sources was performed to improve reliability, validity and consistency of the findings.

10.1136/bmjgh-2023-012125.supp1 Supplementary data

Documents were analysed using the same coding frame after the interviews were completed (January–June 2015), to triangulate the information obtained from interviews. Once the different types of interconnections and links were identified in the literature and interviews, we identified the mechanisms underpinning these relationships, the type of network (defined as ‘issue network’ or ‘policy community’, see Box 1), the type of interaction between actors, and the principles of responsiveness, participation and accountability they affected. For instance, we sought to identify if a collaboration could jeopardise the accountability of the soda tax impact or weighted participation of actors on the debate; or if the participation of those actors might jeopardise the responsiveness of the SSBs tax. Box 1 sets out the definition of policy networks, conflict of interests, type of resources shared, type of interactions considered and types of networks defined for the purpose of this paper.

Box 1 **Definitions of concepts used for the analysis**

**Actors** are all the institutions or persons representing an institution or an organisation that were involved in the policy process. During the policy process, numerous actors participate, though in practice, only those who are members of specific policy sub-systems tend to participate. Different theories use different names to refer to actors depending on their specific role in the policy process. For example: veto players, policy entrepreneurs, or interest groups.^111^

**Power** is defined ‘as an actor’s ability to induce or influence another actor to carry out his directives or any norms he supports’.^112^ Power is the ability to influence others by shaping their preference or the ability to achieve the desired action.^113–115^ For this case study, “the capacity to have an effect on the development of the soda tax”. In this thesis, ‘influence’ or ‘power’ is defined as ‘the capability or ability to accomplish something’.^116^

**Governance** refers to the mechanisms, processes, and institutions through which citizens and groups of actors articulate their interests, mediate their differences, and exercise their legal rights and obligations.^116^ Or ‘the actions and means adopted by a society to promote collective action and deliver collective solutions in pursuit of common goals’.^117^

**Institutions** are organisations, laws, and rules that are central to every political system or area in which policy-making is done.^118 119^ Sometimes, certain institutions have an implicit group of norms of rules rather than an explicit form of them.^120^

**Policy networks:** a set of formal and informal linkages between government and between other actors structured around shared beliefs, interests and resources (as defined in Table 2) for policy making’.^37^

**Conflict of interest:** (COI) arises in circumstances where there is potential for a secondary interest (a vested interest in the outcome – of the programs) to unduly influence, or where it may be reasonably perceived to unduly influence, either the independence or objectivity of professional judgment or actions regarding a primary interest (in this case the program to be delivered).^121^ **Resources could be**

- finance,
- knowledge,
- expertise,
- technologies,
- capacity to mobilize any of these as well as support from members of the network and from those outside it.

**Types of interaction**

**Types of networks**

- **Policy communities are** networks defining the context of policymaking in specific policies where boundaries are clearly defined.
- **Issue networks** are less tight networks with a large number of stakeholders, including academics, interest groups, and experts.

**Principles of good governance**

- **Responsiveness** refers to the policy response to population health needs.
- **Participation** how the actors take part in the decision-making process and how they voice their views, and
- **Accountability** refers to how actors involved in policymaking (government officials, academics, private sector and civil society) are liable to the public.

## Results

On the basis of the stakeholder analysis, the actors involved in the SSB tax were categorised as follows: international organisations (agencies and international civil organisations), government organisations (including different sectors of the government), academics, national civil society organisations (CSOs) and think tanks (TTs); and private-sector organisations, including media actors, private-funded associations, business associations or consortiums, F&BI and marketing firms for soft drinks.

### Types of networks and links identified

**Figure 3:**
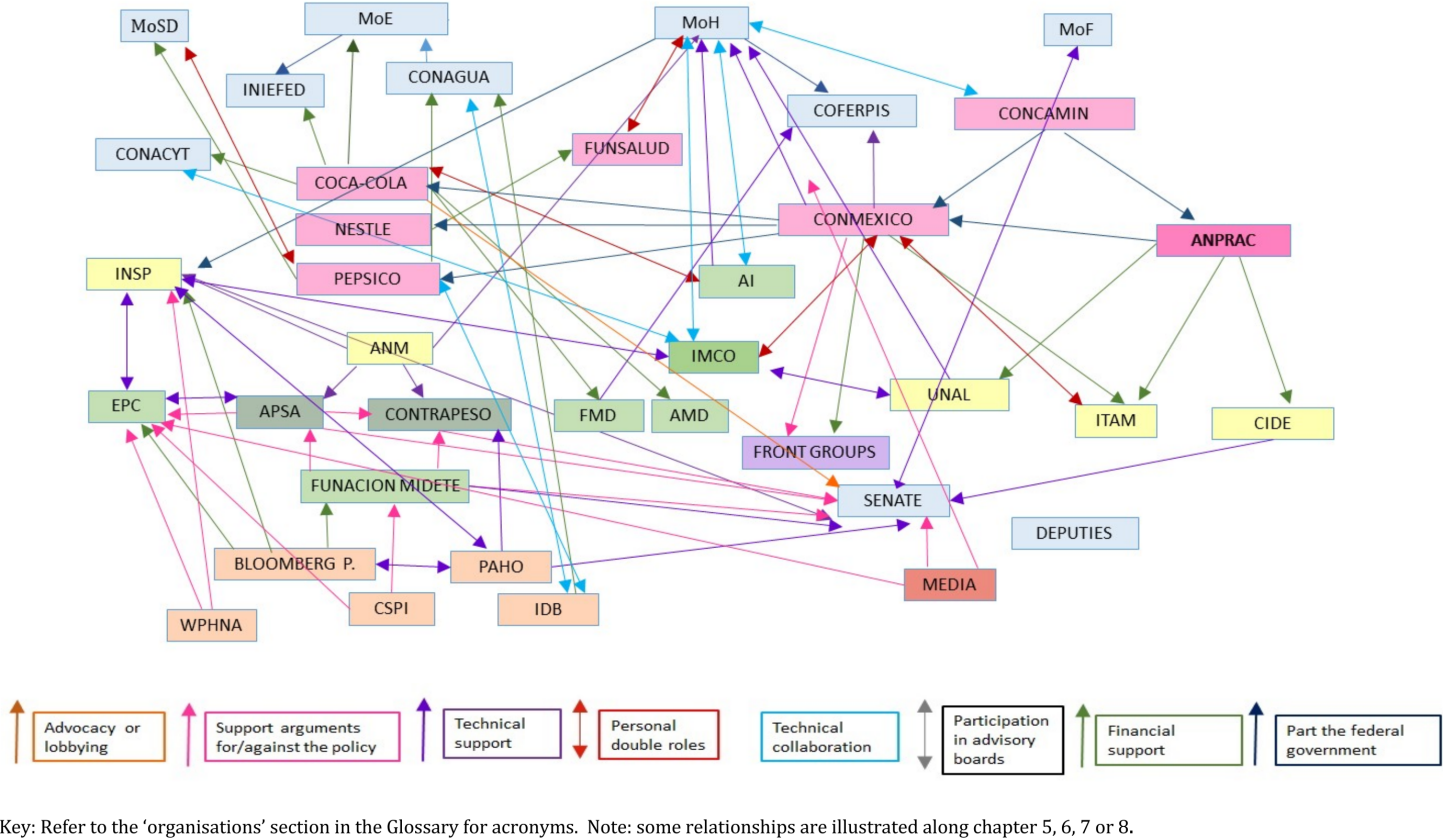
Links identified among actors before, during and after the soda tax.

**Figure 4:**
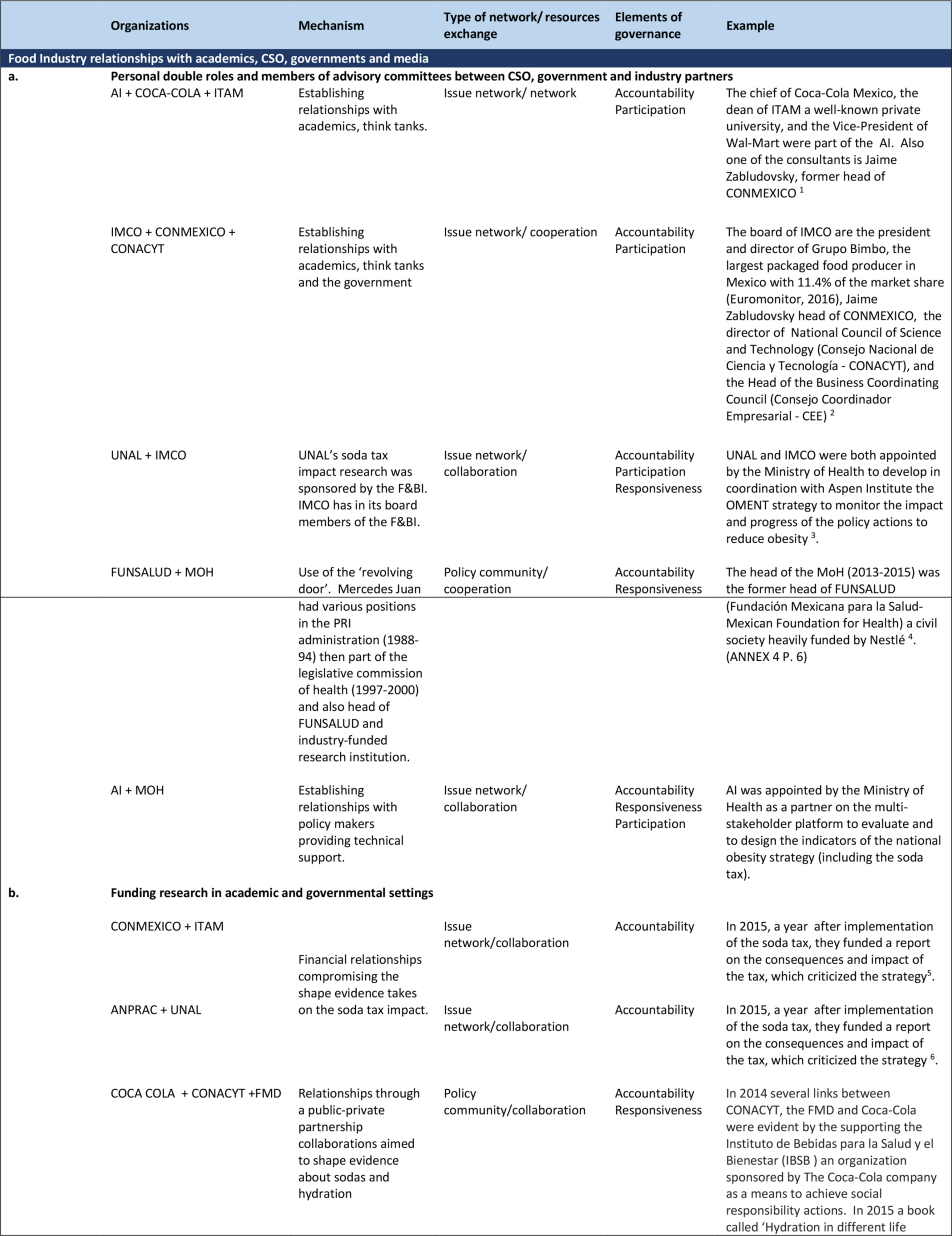

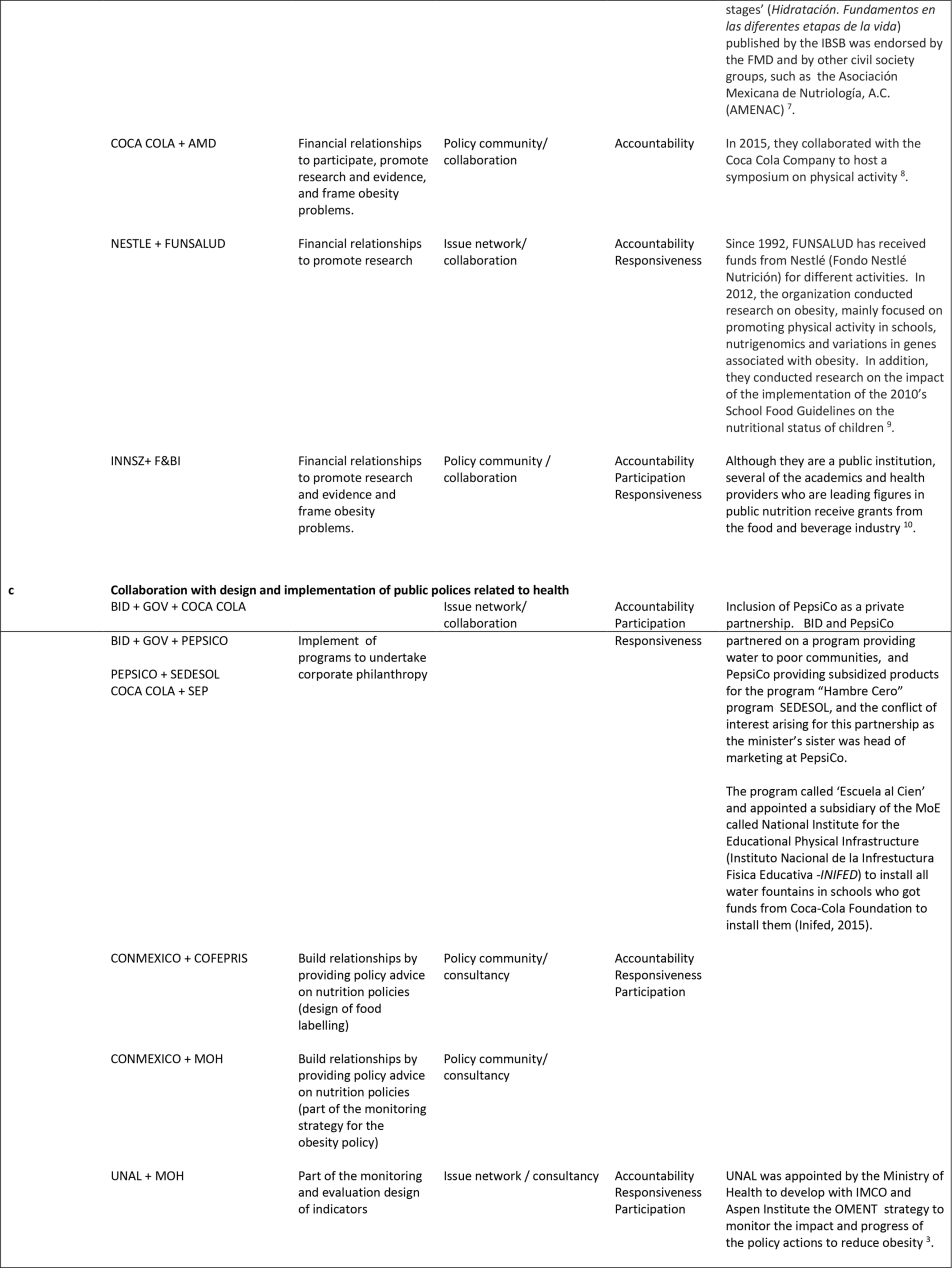

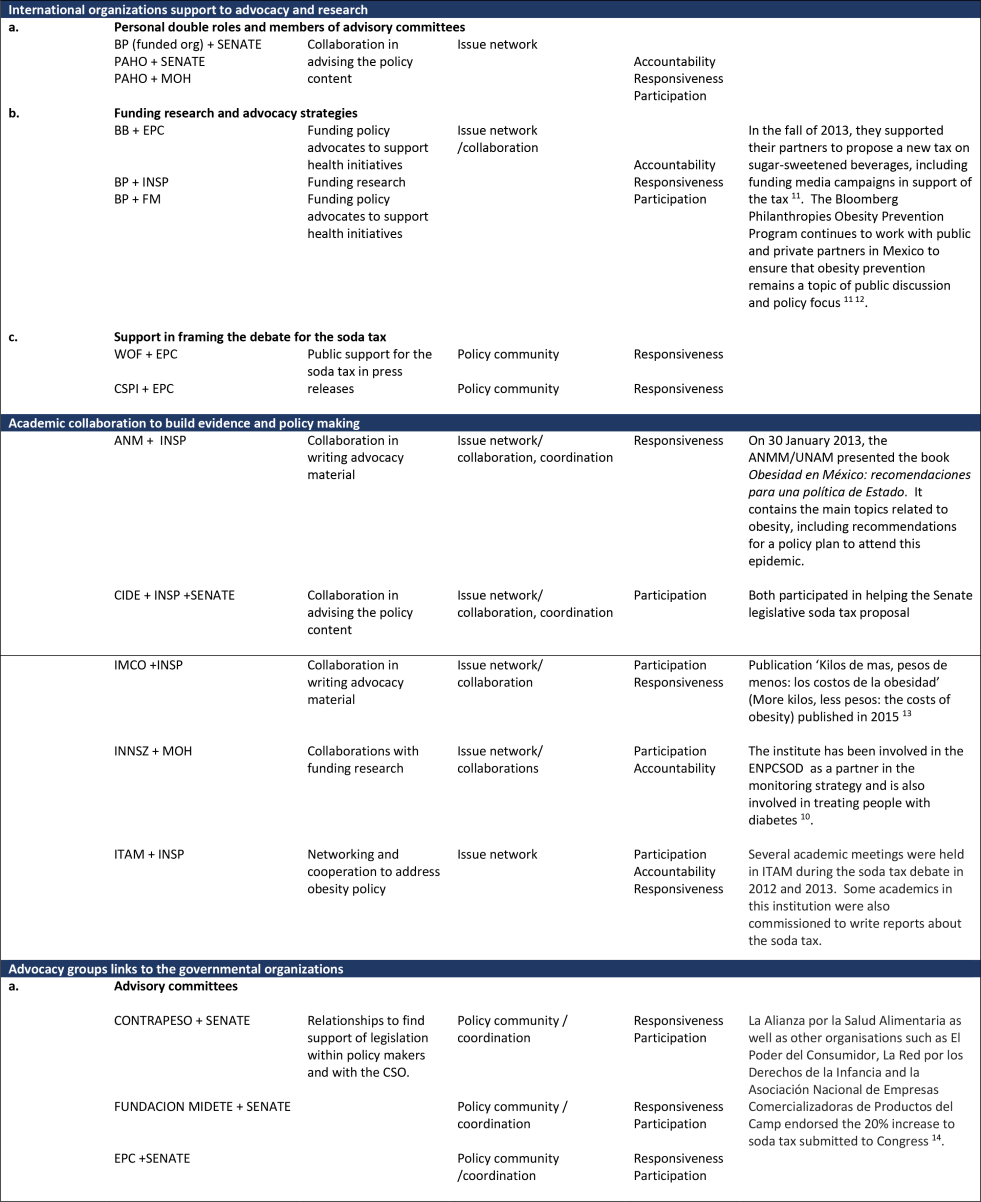
Links identified among actors before, during and after the soda tax. AI, Aspen Institute;CSO, civil society organization; FUNSALUD, Fundación Mexicana para la Salud; IMCO, Instituto Mexicanco de Competitividad; ITAM, Instituto Tecnológico Autónomo de México, UNAL, Universidad Nacional de Nuevo León.

Different types of links found among actors were: (1) individuals playing double roles in two or more organisations, (2) members of advisory panels from different sectors being involved in policy design or advocating for policy changes, (3) financial links between groups and (4) partnerships being entered into a particular purpose, such as advocacy activities or policy implementation or evaluation. A representation of the links and relationships between actors is shown in figure 3, and figure 4 presents examples of relationships identified, the type of resources interchanged and some elements of good governance that might be at stake in those partnerships.

#### Networks and links between the F&BI, civil society and government

Constituency building has been part of the ‘playbook’ of private sector businesses such as tobacco and alcohol companies.^9 46 47^ Establishing relationships with key leaders and policymakers is one way to influence policy.^46–48^ These findings show how some actors representing F&BI companies with the largest market shares of ultraprocessed foods and beverages in the country were related to the OMENT, the body in charge of evaluating the SSB tax policy. Formal participation of these actors in the policy process has been described elsewhere,^49 50^ but the existing relationships that are relevant to the structure and process of the SSB tax policy are discussed ahead.

Three types of formal links were identified between the F&BI and the government: (a) multiple roles of individuals in government and industry positions, including participation on advisory boards; (b) providing financial assistance to government and civil society groups; (c) building partnerships with government agencies for implementing, monitoring or evaluating policies related to food and water provision.

##### Multiple roles of individual actors and ‘revolving doors’

Multiple roles of individual actors, or the ‘revolving door’ concept of individuals changing job roles between public and private sectors, were found. Two policy advocacy organisations, AI and IMCO directors or consultants, held influential roles in three private sector entities: Coca-Cola FEMSA, CONCAMIN and Bimbo (online supplemental material table 2). Both organisations were invited to participate in the MoH’s in the OMENT, to set indicators, goals and outcomes to measure the overall ENPCSOD. For instance, AI’s board included the former head of Coca-Cola Mexico, the Dean of the well-known private university ITAM (Instituto Tecnológico Autónomo de México) and the Vice-President of Walmart. AI consultants included the head of CONMEXICO and former key leader of the NAFTA negotiations.^51^ IMCO had among its board members the Chief Executive of Grupo Bimbo, the largest packaged food producer in Mexico with 11.4% of the market share according to Euromonitor 2016’s report, and the head of the CCE (Consejo Coordinador Empresarial - Business Coordinating Council).^52^

In 2013 Mercedes Juan was appointed as the Secretary of Health. She was the former Executive President of FUNSALUD, a foundation conducting research financed by food corporations such as Coca-Cola, Kellogg’s and Nestlé.^53^ A few members of FUNSALUD board transitioned to be part of the federal government too.^53^ When the SSB tax was discussed, the F&BI and political leaders argued that it was time for all those concerned with the the SSB tax to move forward, away from a ‘confrontation’ with each other, and towards ‘cooperation’ and ‘dialogue’ on other urgent issues.^54^ ^53–55^ Meanwhile, during the inital stages of the SSB tax debate, Mercedes was quiet until an interiview where she said she did not support it. 56 However, in 2014, her postion changed manatianing that the SSB tax was an effective measure to reduce sugar consumption. After the first public discussions on the impact of the SSB tax in 2015, she estabished the OMENT and simultaneously, concerns about conflicts of interest (see Box 1 for definition) of some of its apponted memebrs and withing the MoH were raised by the civil society. Later that same year, Juan was replaced by José Narro, the former head of the Mexican National Autonomous Univesity (Universidad Autónoma de México- UNAM), who released a conflict-of-interest policy for the MoH staff.

##### Financial links with government agencies and funding programs

Financial links were found among organisations involved in the SSB tax discussions. Some of these relationships included government organisations as a third party. For instance, a national programme to improve water and sanitation in poor communities was partially funded by PepsiCo and by the Inter-American Development Bank, as stated in a PepsiCo company report.^57^ This type of collaboration, exchanging information and resource sharing, could be seen as an issue network or the formation of an issue network as it ‘had a funding estimated at $1 million will come from AquaFund, a programme funded by the IDB, PepsiCo Foundation, Swiss Cooperation, and the Austrian government’ to benefit 600 000 people with access to drinking water.^57^

Some links and partnerships between F&BI and CSO were not disclosed to the public during the SSB tax debate. These included links between the Mexican Diabetes Federation and Coca-Cola, Interamerican Development Bank and PepsiCo, Coca-Cola and the National Council of Science and Technology (CONACYT) and the National Institute of Nutrition and the Food and Beverages Institute.

##### Funding academic research

Some academic institutions, both private and public, had funding from the F&BI, following a common strategy used by the F&BI to influence the agenda.^58 59^ The Coca-Cola Foundation sponsored research through CONACYT, the governmental agency that supports research in the country. In 2015, they launched a contest for any scientific project that would improve science and technology and contribute to capacity building in the country (CONACYT, 2015), which some scholars criticised.^60 61^ CONACYT also partnered with the Institute of Beverages for Health and Well-being (Instituto de Bebidas para la Salud y el Bienestar), a research group fully funded by Coca-Cola to sponsor the ‘First Prize in Biomedical Research’.^62^ In 2015, the Coca-Cola Company, through an organisation called ‘Exercise is Medicine’ (Movimiento es Salud) partnered with the Mexican Diabetes Association to have a scientific event called ‘Physical activity for people living with diabetes’. According to Coca-Cola’s annual report, ‘Coca-Cola also provided logistical support for the event’.^63^

After the SSB tax was implemented, the academic institution UNAL’s role as an agency of the obesity policy was compromised. This university was appointed to design the indicators for evaluating the obesity policy, and during this period, received money from the National Association of Soda and Soda Water Producers (Asociación Nacional de Productores de Refresco y Agua Carbonatada—ANPRAC) to publish a report on the impact of the SSB tax just 1 year after the tax implementation. The F&BI, through CONMEXICO and ANPRAC also funded academic work on the impact of the SSB tax published by a group of economists at ITAM. It framed the levy’s effect as minimal ‘[SSB tax] did not significantly reduce caloric consumption […] it only caused a reduction of 6 of the 3200 daily calories consumed by an average Mexican’.^64^ The report mentioned that ‘CONMEXICO did not have any veto power nor influence over the results’, regardless of the narratives used against the SSB tax.^65^

##### Creation of front groups

Several ‘issue networks’ and several ‘policy communities’ (as defined in Box 1) were created during the public discussion about the SSB tax before it was approved in the Fiscal Reform. The F&BI did not publicly disclose that they partnered with health organisations and the Mexican Federation of Diabetes, nor did they publicly announce their support for certain non-government organisation.^66 67^ For example, one group called Sweeten your Life (Endulza tu Vida) was sponsored by the sugar industry (CNIAA)^67^ tried to legitimise itself by including registered nutritionists in its leading team to give recommendations about sugar consumption.^68^ Furthermore, the F&BI participated in sponsoring organisations such as Mexico is Moving (Mexico se Mueve), Active Mexicans (Mexicanos Activos A.C.) and The Centre for Consumer Freedom (Centro para la Libertad del Consumo).^69 70^ The first two organisations were set up to promote physical activity, and the third one was a front group advocating for the right to free choice. Some CSOs known to have partnerships with the F&BI, either front groups or business-interest non-governmental organisations (BINGOS), did not make public any conflicts of interest during the SSB tax debate but refused to be interviewed for this research.

##### Collaboration of the F&BI with the government to design and implement public policies related to health

The F&BI provided either financial or technical support to the government through public-private partnerships (PPPs). During the SSB tax debate, the F&BI partnered with government organisations to implement parallel policy actions such as providing drinking water in schools (eg, CONAGUA and INIFED) or providing technical support to the OMENT.^71–73^ Other relevant PPPs at the time were agreed between CONAGUA, the Mexican water regulatory body, the World Bank and Coca-Cola FEMSA to provide water to selected poor Mexican communities through a programme called PROMAGUA. Coca-Cola obtained seven government concessions to provide water with them to the poorest communities, avoiding any further payment to the authorities for the water supply.^74^ Also, simultaneously with the tax implementation, in 2015, the PepsiCo Foundation signed an agreement to support the International Youth Foundation with a training programme called ‘Key to the Future’ aimed to prepare young students from the 39 campuses of the National College of Professional Technical Education (Colegio Nacional de Educación Professional Técnica—CONALEP) in the State of Mexico for integration into the labour market.^75^

In mid-2016, the Mexican president inaugurated the Coca-Cola Innovation and Development Centre in Chiapas and recognised it to be an effort to improve the country’s economy.^72 76^ Parallel to the events, and all the emerging collaborations between the SSBs companies and the federal governments, the president signed an agreement with the industry to dodge any bill aiming to increase the SSB tax during his mandate. This same year, Congress did have a policy debate about increasing the tax-supported by pro-tax coalitions. Reports of a spy web to health activists at the time was a matter of concern internationally, and investigations implied it was a potential intervention of the federal government.^77^

#### Networks and links between international organisations, CSOs and academics

Several ways on how international organisations were involved in the SSBs tax policy were identified, such as providing financial or technical support or framing the debate.

##### Funding research and advocacy strategies

In Mexico, Bloomberg Philanthropies (BP) provided technical and financial support for ‘an obesity prevention programme’ with a 10 million grant for a 3-year programme (BP, 2016). They supported a public-lobbying firm (TT) called PoliThink, an academic institution, the National Institute of Public Health^78^ and a CSO, El Poder del Consumidor and called all of them ‘partners’.^79^ The simultaneous support to different organisations started as an ‘issue network’ aimng to build a strategy that led to the SSB tax implementation^79^ and shifted the power of actors involved in policy debate and process. As mentioned in BP’s website, for the first time the financial power of Mexico’s SSB industry faced a serious challenge.^80^

##### Support in framing the public debate

According to a BP report, the success of the SSB tax was achieved through their strategy, which centred on two activities: paid and earned media campaigns and formal lobbying; both of which leveraged scientific evidence and a rigorous understanding of the political context.^81^

In addition, other international organisations, such as the Pan American Health Organization (PAHO) and World Obesity Federation, were strong advocates for the pro-tax groups and repeatedly disclosed their support for the SSB tax during the policy discussion.^80 82^ The World Public Health Nutrition Association and Centre of Science for the Public Interest civil society groups also supported by issuing several press releases.

BP paid for media campaigns designed and promoted by Mexican civil society groups with support of academics. For instance, Dr Robert Lustig, a well-known international expert in obesity, was invited to talk to the media. In May 2013, he participated in a forum called ‘The Sugar Pandemic: Policy vs Politics’, speaking about the harm caused by sugar and SSB consumption to people and how the F&BI uses its influence to block effective public health policy. The overall paid-for efforts resulted in nearly 800 media stories on obesity.^81^ This meant that the coalition of actors supporting the SSB tax was well funded for the first time and had wide support from strategic groups and public-interest lobbying firms. As a representative of BP mentioned, funding ‘levelled the playing field’.^63^ Academics were subsequently able to make their positions clearly heard in newspapers and blogs and gained credibility among citizens and policymakers.

Most of the networks between CSOs were built during the SSB policy debates, such as ContraPESO, a collaboration of several CSOs. In contrast, some of them already existed, such as Alianza por la Salud Alimentaria. The former is classified as an issue network and the latter a policy community.^83^ The private sector organisations ranged from established coalitions such as CONAMIN corporate consortium to newly formed non-governmental organisations called BINGOS.^84^

#### Networks and links between academics and scholars

Two main collaborations were identified as key aspects of the agenda-setting for the SSB tax. The National Insitute of Public Health (Instituto Nacional de Salud Pública -INSP) engaged in collaborations to gain support for the policy. First, INSP engaged with the National Academy of Medicine (Academia Nacional de Medicina) and published a book titled ‘*Obesidad en México: Recomendaciones para una política de Estado*’ (Obesity in Mexico: Recommendations for a State Policy) and presented it in January 2013. The book was used repeatedly in academic and public discussions on the SSB tax and other policies as it provided some suggestions on the regulations needed to improve public policies for obesity and diabetes.^85 86^ A second key collaboration between the INSP and IMCO was capitalised by publishing a report that mapped policies selected in the strategy and compared them to the benchmark indicators produced by the World Cancer Research Fund International. This resulted in the publication ‘*Kilos de mas, pesos de menos: Los costos de la obesidad*’ (More kilos, less pesos: The costs of obesity). This study estimated the social costs of obesity, including expenditures on medical treatment and productivity losses due to premature mortality and work absenteeism. It also reviewed the public policies that had been adopted to address this problem and compared their design with international benchmarks to provide some recommendations.^87^ The link between these two organisations identified through this collaboration also disclosed that IMCO was in a potentially compromised position, as the executive board included members of the F&BI industry who opposed the tax; while the INSP was one of the allies in promoting the SSB tax throughout the process. Nevertheless, IMCO stopped any participation in the SSBs debates after the publication.

#### Advocacy groups and CSOs link to government officials

The most relevant relationship identified between pro-SSB tax advocacy groups and the government were the relationship between a Senator called Marcela Torres, a Tink Tank (TT) and a civil society coalition (Fundación Midete, Polithink, ContraPESO), who together designed the SSB tax legislative proposal in 2012.

Our data did not show any direct link between the federal government and the pro-tax civil society groups during the policy debates. However, PAHO led a multidisciplinary group that included MoH, civil society and academia members.^82^

## Discussion

Given the complexity of the policy context Mexico had at the time of the SSB tax policy, the networks found resemble ‘issue networks’ and ‘policy communities’, some of them more exclusive and influential than others, which were formed during the SSB tax discussion and formulation and were a key aspect for achieving the policy change. Complex relationships and links were identified, including relationships between companies and the government, civil society groups and international organisations, academics and industry, TT and industry consortiums, F&BI and ministries, academics and activists and civil society and legislators. Some were advisory relationships, and some were financial relationships, collaborations, consultations or even situations of nepotism in high-level positions.^88 89^

Our findings suggest that some relationships and links contributed to and influenced the SSB tax, and to it being set at 10% instead of 20% as initially proposed by academics. This case study exemplifies the value of (1) not only drawing on general insights from policy theories but also (2) going deeper to identify what networks exist and why.^32 33 90^ The policy debates and outcomes depend on how the relations among interested actors developed. While it is an organic part of the process for actors to interact in policymaking, there are other less visible relations and links that need further exploration to understand the politics behind the process and corporate power and the factors facilitating or undermining this power. For instance, the close relationship Coca-Cola has had with the federal government for decades continues, despite the changes in administration; versus the empowered civil society groups and health advocates that emerged from this experience working closely with an international partner, BP, and that continue to work in other food policy issues.^55 61 91^

New coalitions were built among civil society, academics, TT and international organisations in the field of nutrition policymaking in Mexico. As suggested by Huang *et al*, obesity coalitions, among other strengths, can control media messages rather than letting these messages be controlled by the industry or diluted by uncoordinated organisations.^92^ For example, academic collaborations were key to building a narrative to frame obesity and sugar-sweetened drink consumption as a problem and frame its potential policy solutions.^50^

Support from international actors provided to national civil society groups and academics helped change the power dynamics during the debate. It supported media advocacy actions and the involvement of TT in the debate.^55 61 91^ Previously, support from international organisations had been relatively low for civil society groups in Mexico advocating for nutrition policies. The shift in power to the public-interested groups served as a leverage point for advancing and implementing the policy.^91^ Results show that such international support is rare, as the nutrition and health sectors in Mexico, including CSOs and related academic groups, are heavily influenced by the F&BI.^93^ Until the SSB tax emerged as a policy option, the participation of the F&BI or the links between them and the government were not perceived as a conflicting interest with their fiduciary duties, or a problematic influential relationship. It continues to be ignored or denied as such by some state policy actors, especially when considering the existing and emerging public-private partnerships (PPPs) between the F&BI and government agencies to address health, nutrition and physical activity programs.^94 95^

The government and F&BI links and collaborations, as those identified here, should be a matter of public concern, despite the advances in the public health agenda Mexico has had in recent years. As our results show, policymaking is embedded in a conflicted environment where the F&BI has high influence and power within the political structures.^55^ The close relationship between the F&BI and the government endangered the primary aim of the SSB tax, namely, protecting health. The identified links highlight the normalisation of connections among actors that go beyond common interests and have competing fundamental aims and goals towards health and other objectives, thereby jeopardising any attempt towards public health solutions for obesity, as other case studies have found.^96^ The power dynamics among those relationships are and continue to be monitored closely by public health interested groups in the country. This research provides evidence of how political science offers insights into relationships that shape public policy.^97^

PNA as a lens to explore Mexico’s SSBs was useful in identifying the opportunities and constraints during the process. At the same time, it might touch on various actors’ sensibilities involved in the analysis. However, the public domain of such relationships has shown to enable public health interest into the agenda, as shown in other case studies using theories on agenda setting.^33 98 99^ Although the PNA is described as ‘an analytical toolbox rather than a theory’,^83^ and has been criticised owing to its limited theoretical basis, it has been incorporated into other frameworks,^100–102^ and has emerged as an essential analytical tool to research governance structures, power dynamics^103^ and multistakeholder partnerships in food, alcohol policies^104 105^ and tobacco policies.^106^

This approach considers networks as a type of governance that provides insights into participation, responsiveness and accountability principles.^33 38 107^ Using networks approaches comes from dissatisfaction with structural–functional analyses and the search for alternative ways to interpret social action and understand the implication in a set of relationships.^108^ In this study, the participation of different stakeholders was exemplified by the rhetoric used by federal governments and other actors on the relevance of an inclusive and multistakeholder approach to obesity policy. Responsiveness to the policy and accountability of the process were concepts not reflected in the narratives. However, they were noted as elements of concern when analysing links between stakeholders and the role the private sector played in the evaluation and monitoring of the obesity policy. Other examples included contrasting arguments about the formal representation of actors in the discussion tables, the lack of indicators to evaluate the SSB tax in the obesity monitoring strategy and the calls for transparency and accusations of conflicts of interest during the design of the ENCSOD and the OMENT platform.

Accountability involves one actor answering to another actor or group of actors, who can assess how well the former fulfils their requirements to achieve specific goals.^109^ In this case, the accountability function was performed by civil society actors. Despite calls for accountability by civil society and other supporters of the tax, the F&BI was legitimised by the government throughout the process, and the conflict of interest between the industry’s aims and public health goals was overlooked by policymakers. The concentration of power remained in the corporate sector. The increasing PPP between F&BI and goverment, represented a ‘horizontal cooperation’ between them, where the distribution of power was similar between actors.^110^ Civil society and the legislative power had cooperated closely to draft the bill when the discussion of the tax started in early in 2012. However, this was not the case for civil society’s relationship with the federal government, as there was minimal interaction, it was an ‘asymmetric bargaining’ interaction where the distribution of power had a hierarchical element, as the power remained in the dominant group, namely the government, who for instance, changed the final tax to 10%, despite civil society groups and academics suggesting to have at least a 20% excise tax.^110^

### Limitations

One of the main limitations of this research is that it does not compare the soda tax in Mexico with a similar situation in other countries. One challenge faced during the interview period was the lack of participation of some key actors, mainly from not only the F&BI but also some academics and a representative of the media. The use of PNA as a lens to explain the politics of the SSBs tax considers concepts useful to describe our findings but that gathers different conceptualisation of the role of institutions and actors in policy from the political science discipline.

## Conclusions

Our findings provide empirical evidence on how relationships and links between different stakeholders, publicly disclosed or not, contributed to and influenced the agenda setting and policy formulation of the SSB tax. Public arguments provided by corporate actors about a positive or negative position for the SSB tax may be a publicly acceptable justification for their political interests. However, publicly unrecognised interests, private relationships, sources of support and double roles can offer a better understanding of policy actions. Mapping the actors involved in the policy and analysing the type of networks provides further understanding of actors’ views and whether they are potentially compromised. It also raised key questions about conflict of interests, accountability (who is accountable to whom and through what mechanisms), representation and power imbalance in the policy process. This work provides an opportunity to examine both the contextual issues that drove the SSB tax in a particular direction and enabled it to happen and the issues that restricted its further development.

## Data Availability

Data are available upon reasonable request.
