# Supplementary material for: Policy networks and competing interests in the development of the Mexican sugar-sweetened beverages tax
Source: BMJ Glob Health. 2023 Oct 9;8(Suppl 8):e012125. doi: 10.1136/bmjgh-2023-012125 (PMC10565318; doi:10.1136/bmjgh-2023-012125)
Supplement: Supplementary data [file bmjgh-2023-012125supp001.pdf]

Supplementary material

Themes for analysis used for interviews and documentary analysis

To understand the contextual factors that facilitated the development of the soda tax
Barriers perceived of the context
Change of government
Environmental conditions
Fiscal reform
Government OR Institutional issues
Obesity increase
Opportunities perceived of the context
Social norms and perceptions (cultural factors)

To analyse the policy-making process surrounding the soda tax
Drivers for the policy change
Agenda setting and timing
Actions involved on shaping the policy design
Dialog and engagement of the actors
Means to evaluate, monitor and (or) modify the policy
Perceptions of the implementation
H2O issues

To examine stakeholders position and how each one attempted to influence the soda tax Implementation
Approaches to influence perception of the population
Credibility of the proposal
Dominant policy narratives
Engagement and involvement of stakeholders
Framing the policy
Framing the problem
Participation (actors beliefs and values)
Perception of the obesity policies
Power of the actors

To document the main policy outcomes of the stakeholders after the policy change
Acceptability of the policy change
Accountability after the soda tax
Changes made to overcome the outcome
coercive
conflict of interest
corruption
governance
Main outcomes of the soda tax
Regulatory, monitoring and voluntary actions emerging as collateral actions by different actors
right to health
social responsibility

Definition of themes and sub-themes identified in the documentary review

Theme	Sub-theme	Definition
**Approaches to influence perception of the policy change**		Refers to the arguments used by different actors to try to influence opinion. Could be used to influence consumers opinion, policy makers opinions or other actors such as academics or civil society members.
	Multicausality of obesity	It includes positioning the issue by itself or accompanied by other issues.
	Calls for action	This are the main strategies different actors have used to increase support to their own benefit. It includes calls for the government to regulate, public demonstrations (against/in
	Marketing strategies	Promotions, discounts, expanded publicity of products. Use of marketing to promote messages.
	International actions	some sources referee to what actions have been recommended or done internationally to credit or invalidate the policy change.
	consumption (both negative and positive)	This framing refers to positive and negative implications soda tax and how such perceptions were framed throughout time (before, during the debate and once the tax was
	Coalition building	Coalition building is one of the main strategies mentioned in literature and in our data a s a strategy used by different actors to empower their position.
	Social responsibility	Emerging actions by different stakeholders throughout time are described as part of compromising themselves with a cause occurring at the time of the soda tax (either directly with obesity, soda consumption or other contextual factor such as water availability, drinking water, or environmental issues).
	Right to health and healthy environments	Arguments about the right to have access to healthy foods and limited access to unhealthy foods, the right to accurate information, health and water access.
**Changes in law and reactions of actors**		It includes the main foresight of different actors of the forthcoming policy changes or the current policy changes occurring in the country and how are they related to their own means and aims.
**Credibility of the soda tax**		All the expressions of the implications of the soda tax
	Credibility of stakeholder actions	Expressions on how believes what will work and how will work. This theme reveals the links and supporting actions between actors creating networks.
	Effective or Ineffective	Expressions about the effectiveness or ineffectiveness soda tax, the implications (before it was implemented) and its effects (after implementing it).
	Side effects	Highlighting other effects that the soda tax has, including adaptations of the SSB companies to the new context, changes in strategies and change of paradigms from the civil
**Evidence and sources of credibility**		Evidence seems to have competing arguments around the effective or ineffective role of soda tax, and to the type of reports presented publicly. The framing of certain documents highlight the position of the authors about the failure or success of the policy measure.
**Ties between actors**		This theme is to disclose how networks were built around actors who have played a role in the soda tax imitative.
	Civil society- Academics	All type of interactions between civil society members (alone or in group) to the academic groups (or individuals).
	Civil society- Food & Beverage industry	All type of interactions, collaborations and endorsements between civil society members and F&B industry members (or groups)
	Civil society- Government	All type of interactions, collaborations and endorsements between civil society members and Government members (or groups).
	Civil society- International agencies	All type of interactions, collaborations and endorsements between civil society members and international agencies.
	Civil society-Journalists/ Media	All type of interactions, collaborations and endorsements between civil society members and journalists or media (companies or individuals).
	Academia- Government	All type of interactions, collaborations and endorsements between academics and government members.
	Academia- Food & Beverage industry	All type of relationship between academics and members of the food and beverage industry or any of its endorsed front groups.
	Academia- Journalist/Media	Interactions or endorsement of journalists or media (or lack of it) with civil society members.
	Government-Food & Beverage	All type of interactions, collaborations and endorsements between the government (federal or legislative power) with food and beverage industry's members.
	Government- Journalist/Media	Interactions regarding soda tax issue between government members and media members or journalists in regards the soda tax.
	Journalists/Media- Food & Beverage industry	Interactions regarding soda tax issue between food and beverage industry's members and the media or journalists.
	Governmental relationships between powers (legislative and federal gov)	Looks at the dynamics between actors and sectors (legislative and federal) members before, during and after the soda tax passed.
**Framing the problem**		The main argument used to support the thesis about an issue and that ha s been identified as a public problem, according to Laswell policy cycle.
	Obesity in Mexico is a public Health problem	Public health is defined as "the science and art of preventing disease, prolonging life and promoting health through organized efforts and informed choices of society, organizations, public and private, communities and individuals". By framing obesity as a public health problem, obliges a response from all the mentioned sectors and people.
	Economic implications of obesity	Highlighting the economic impact of obesity is the most used narrative among academics and politicians, but is also used in some documentations of the private sector.
	Excess intake of calories & energy balance are the causes of obesity	Highlights the multifactorial causes of obesity, to avoid pointing out a single issue such as sugar or soda
	Soda consummation	Highlights the excess consumption of soda in Mexico, and as such the need to address it by the soda tax.
	Taxing SSBs	Highlights the implications and the problems emerging and related to taxing sodas.
	Sweetened Beverage Industry	Highlights how the main problem is related to the power and the position the soda industry has in the country.
